# Field Evaluation of the New Rapid NG-Test^®^ SARS-CoV-2 Ag for Diagnosis of COVID-19 in the Emergency Department of an Academic Referral Hospital

**DOI:** 10.3389/fpubh.2022.840984

**Published:** 2022-04-25

**Authors:** Kalliopi Avgoulea, Maria-Ioanna Beredaki, Sophia Vourli, Maria Siopi, Nikolaos Siafakas, Spyros Pournaras

**Affiliations:** Clinical Microbiology Laboratory, “Attikon” University Hospital, Medical School, National and Kapodistrian University of Athens, Athens, Greece

**Keywords:** NG-Test^®^ SARS-CoV-2 Ag, rapid diagnostics, antigen test, point-of-care, COVID-19, emergency department, lateral flow immunoassay

## Abstract

**Background:**

As the COVID-19 pandemic resurges affecting large numbers of patients, rapid, and accurate diagnosis using point-of-care tests is very important.

**Objectives:**

To evaluate the NG-Test^®^ SARS-CoV-2 Ag (NG-Test) immunoassay for qualitative detection of SARS-CoV-2 antigen in nasopharyngeal (NP) and oropharyngeal (OP) samples compared with RT-PCR, in patients attending the Emergencies of an academic referral hospital.

**Methods:**

All adult ambulatory patients presenting to the Emergencies of “Attikon” University hospital (Athens, Greece) within three consecutive hours per day between December 2020 and March 2021 and for whom SARS-CoV-2 PCR testing was requested were included. Two NP and one OP samples obtained from each participant were analyzed to determine the diagnostic performance [sensitivity, specificity, positive/negative predictive values (PPV/NPV)] of the NG-Test (NP/OP swabs) in comparison to the reference RT-PCR (NP swab).

**Results:**

Overall, 134/263 (51%) patients tested were RT-PCR positive, whereof 108 (overall sensitivity 81%, 95% CI 73–87%) were NP NG-Test positive (PPV 99%, NPV 83%) and 68 (overall sensitivity 51%, 95% CI 42–59%) were OP NG-Test positive (PPV 100%, NPV 66%). The test's specificity (95% CI) was 99% (95–100%) and 100% (96–100%) for NP and OP swabs, respectively. The assay's sensitivity (95% CI) for high viral load (C_t_ ≤25) was 99% (92–100%) and 71% (60–81%) for NP and OP swabs, respectively.

**Conclusions:**

NG-Test using NP swabs detected almost all patients with high viral loads, showing satisfactory performance as a point-of-care test for NP samples obtained from patients with acute infection.

## Introduction

Two years after the first reported case of SARS-CoV-2 infection in Wuhan, China (1), COVID-19 remains an ongoing global pandemic (2). Despite the vaccination strategies and other mitigation measures implemented regionally, exponential resurgence has been repeatedly reported worldwide (3, 4).

The gold standard for COVID-19 diagnosis is the direct detection of SARS-CoV-2 viral RNA using nucleic acid amplification techniques (real-time RT-PCR) (5, 6). However, PCR testing during periods of high COVID-19 incidence has been recently questioned due to the quite long turnaround time, which strongly influences the function of Emergency Departments (EDs). Further limitations, particularly for low-resource settings, are the need for expensive equipment and reagents as well as the requirement for specialized biocontainment laboratories, operated by highly trained personnel (7). To address this reality, simple and rapid antigen detection tests (RADTs) are recommended by the World Health Organization (WHO) for use in the community for primary case detection, contact tracing and outbreak investigations (8).

Based on these grounds, the aim of the current study was the clinical evaluation of the NG-Test^®^ SARS-CoV-2 Ag (NG-Test), a novel lateral flow immunoassay for the rapid detection of SARS-CoV-2 antigen (nucleocapsid protein), in both nasopharyngeal (NP) and oropharyngeal (OP) samples of ambulatory patients who visited the ED of our hospital, in comparison to real-time RT-PCR being implemented for COVID-19 diagnosis in our setting.

## Materials and Methods

### Study Design

The study included all adult ambulatory patients who presented to the ED of “Attikon” University hospital, Athens, Greece, within three consecutive hours per day between December 2020 and March 2021 and for whom SARS-CoV-2 PCR testing was requested by the clinician in charge, without any selection criteria.

A total of three respiratory specimens (two NP and one OP swabs) were collected from each participant after giving informed consent. One NP and one OP collection were screened by the NG-Test (NG-Biotech Laboratories, 35480, Guipry, France), as recommended by the manufacturer. Briefly, two test devices labeled with the patient's name and marked with a distinctive N (for NP) or O (for OP) sign, were placed on a clear and flat surface. After specimen collection, the swabs were immediately unloaded by rotation in the extraction buffer, while squeezing them through the extraction tube; before removal, they were firmly squeezed against the upper tube walls. Immediately after the extraction, a dropper cap was attached to the extraction tubes and 3 drops (~120 μL) of the extracted sample were transferred to the S/R well of the test cassettes. A timer was set and the results were read out visually after incubation at room temperature for exactly 15 min. Low-intensity lines in the test area, in presence of a positive red line control, were interpreted as positive results.

According to the protocol implemented in our setting for COVID-19 molecular diagnosis, a NP swab mixed in viral transport media was immediately transported to the Clinical Microbiology laboratory of our hospital, which serves as the referral COVID-19 diagnostic center for the 2nd Regional Health Authority of Greece. The RT-PCR test that was routinely applied in our laboratory during the study period and was used as the reference method for comparison, was the Certest Viasure SARS-CoV-2 kit (CerTest Biotec, Spain), performed on a Rotor-Gene Q thermal cycler (Qiagen) following the manufacturer's instructions. PCR cycle threshold (C_t_) values ≥35 were considered negative, while positive C_t_ values were recorded. The laboratory personnel were blinded to the identity of patients that were tested by immunoassay during their ED stay and the RADT result.

The study protocol was approved by the Institutional Review Board and Bioethics Committee of “Attikon” University Hospital (ref. number EBΔ 662/30-11-2020).

### Data Analysis

The sample size was determined by our target to test at least 100 prospective RT-PCR positive samples, as it is recommended by the WHO for diagnostic sensitivity determination (8). Medians and interquartile ranges (IQR) were calculated for continuous variables, while frequencies and percentages were calculated for categorical parameters. Performance characteristics, including sensitivity, specificity and positive/negative predictive values (PPV/NPV), for antigen testing were assessed using two-by-two tables and the respective two-sided 95% confidence interval (CI) was estimated. Categorical agreement between RT-PCR and NG-Test was estimated and its strength was assessed by calculating the Cohen's kappa coefficient (κ). All data were analyzed using the statistics software package GraphPad Prism, version 8.0 for Windows (GraphPad Software, San Diego, CA).

## Results

Overall, 263 unique patients were enrolled in the study, whereof 148/263 (56%) were men with median age 50 years (range, 18–89; IQR, 27). The majority of patients exhibited one or more COVID-19-compatible symptoms and sought medical care for the first time (212/263; 81%) or were previously diagnosed (30/263; 11%); most of them (235/242; 97%) presented to the ED within 7 days from symptoms onset. The predominant symptom was fever (188/242; 78%), followed by cough, headache, dyspnea, diarrhea, weakness, myalgia, pharyngodynia, anosmia, ageusia, emesis, and rhinorrhea. A small proportion of patients (21/263; 8%) was asymptomatic and visited the ED as close contacts of a symptomatic patient (Table 1).

**Table 1:** Demographic data of the study population.

**Patients'**		**No of patients**
**characteristics**		**(*n* = 263)**
Sex	Male	148 (56%)
	Female	115 (44%)
Age (years) [median (range, IQR)]	50 (18–89, 27)
Asymptomatic		21 (8%)
Symptomatic	First-visit	212 (81%)
	Previously diagnosed	30 (11%)
Symptoms^*^	Fever	188 (78%)
	Cough	98 (40%)
	Headache	37 (15%)
	Dyspnea	31 (13%)
	Diarrhea	29 (12%)
	Weakness	28 (12%)
	Myalgia	24 (10%)
	Pharyngodynia	21 (9%)
	Anosmia	14 (6%)
	Ageusia	13 (5%)
	Emesis	12 (5%)
	Rhinorrhea	3 (1%)
Presentation to ED within the first week of symptom onset^*^		235 (97%)

* *Percentages were calculated based on the number of symptomatic patients (n = 242). ED, emergency department*.

In total, 134/263 (51%) patients were tested positive by RT-PCR, with a median C_t_ value 25 (range, 13–34; IQR, 8). Among them, 128/134 (96%) were symptomatic, while six were asymptomatic. 108/134 (81%) patients had a positive result in the NG-Test when NP swabs were tested resulting in sensitivity (95% CI) of 81% (73–87%) and NPV 83%, while the test specificity (95% CI) was 99% (95–100%) and PPV 99% (Table 2). The agreement between RT-PCR and NP NG-Test was 90% with κ = 0.795 (95% CI 0.724–0.867) representing strong agreement. On the other hand, only 68/134 (51%) patients had a positive NG-Test result when OP swabs were used, resulting in sensitivity (95% CI) of 51% (42–59%) and NPV 66% [specificity (95% CI) was 100% (96–100%) and PPV 100%] (Table 2). The agreement between RT-PCR and OP NG-Test was only 75% with κ = 0.503 (95% CI 0.413–0.593) demonstrating weak agreement.

**Table 2:** Test characteristics (mean, two-sided 95% confidence interval) of NG-Test^®^ SARS-CoV-2 Ag using nasopharyngeal (NP) and oropharyngeal (OP) samples (each of 263).

	**NP**	**OP**
Overall sensitivity	81% (73–87%)	51% (42–59%)
Specificity	99% (95–100%)	100% (96–100%)
PPV	99% (94–100%)	100% (93–100%)
NPV	83% (76–88%)	66% (59–73%)

*PPV, positive predictive value; NPV, negative predictive value*.

**Figure 1:**
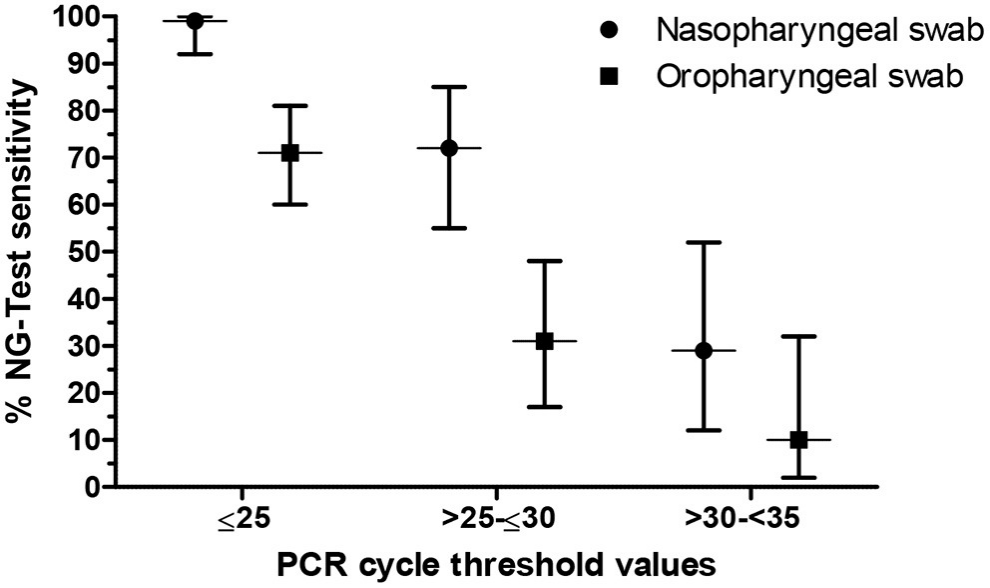
Sensitivity of the NG-Test^®^ SARS-CoV-2 Ag (NG-Test) in respect to the viral load of clinical specimens. Vertical error bars represent the 95% confidence interval.

Given that the overall sensitivity is strictly dependent on the distribution of C_t_ values obtained in the RT-PCR assay within the population of specimens (9), a sub-group analysis was performed and the sensitivity was recalculated in a C_t_-dependent manner. In particular, based on the C_t_ cut-off value, samples with C_t_ ≤ 25 were designated as high viral load, C_t_ >25- ≤30 as intermediate and C_t_ >30- <35 as low viral load (10). The corresponding C_t_ values and sensitivities are shown in Figure 1. Regardless of the nature of the clinical specimen, a significant difference in the performance of the antigen assay was found in patients with different viral loads (*p* < 0.0001). Namely, the sensitivity (95% CI) of the NG-Test when NP swabs were tested was 99% (92–100%) for specimens with a high viral load, whereas for samples with an intermediate and low viral load, the sensitivity was calculated to 72% (55–85%) and 29% (12–52%), respectively. Accordingly, the sensitivity (95% CI) of the antigen test when OP swabs were used was 71% (60–81%), 31% (17–48%), and 10% (2–32%) for samples with a high, intermediate, and low viral load, respectively.

The moderate NPV of NP NG-Test was attributed to a total of 26 false-negative results; one (4%) came from a patient with high viral load, 10 (38%) from intermediate and 15 (58%) from low viral loads. Of the 26 false-negative samples, 14 (54%) were collected from patients being diagnosed from 4 to 14 days earlier, while 12 (46%) were collected from symptomatic, first-visit patients with C_t_ ≥27. The OP screening showed 66 false-negative results; 22 (33%) from patients with high viral loads, 25 (38%) intermediate and 19 (29%) low viral loads. Twenty-three (35%) false-negative OP samples were collected from patients originally diagnosed within 2 weeks prior to ED presentation, while 43 samples (65%) from first-visit symptomatic patients, 17 of which (40%) were from patients with high viral loads, 16 (37%) intermediate and 10 (23%) low viral loads.

## Discussion

The appearance of novel variants of concern, such as the recently designated by WHO Omicron variant, known as B.1.1.529 (11), along with the continuous strain of healthcare systems globally and the need to rapidly diagnose in order to contain the spread, have aroused the interest of the scientific community toward rapid, accurate, cost-effective, and point-of-care techniques. In the framework of our study, the performance of the NG-Test in NP and OP samples was assessed compared to RT-PCR, presenting an overall sensitivity of 81 and 51%, respectively, and a high specificity of 99–100%. Considering that PPV is strongly dependent on the disease prevalence in the study population (12), NG-Test had an excellent PPV (99% in NP vs. 100% in OP samples) within a population with 51% COVID-19 prevalence.

In fact, the performance of the NP NG-Test met the WHO's minimum requirements for RADTs (≥80% sensitivity and ≥97% specificity) for COVID-19 diagnosis (8) being in agreement with the manufacturer's NP performance data (sensitivity 83% and specificity 100%) (13). It also displayed a significant agreement of 90% (κ = 0.795) with the RT-PCR. Furthermore, according to a recent retrospective case control study evaluating the performance of six RADTs in NP samples, a previous version of the NG-Test showed high specificity (98.5%), similar to our results, but much lower overall sensitivity (32.3%), than that in our findings (81%) (14). It should be noted that different antibodies were used in the current version of the NG-Test.

As for the OP swabs, the estimated sensitivity (51%) and level of agreement with the reference methodology (75%, κ = 0.503) showed moderate to weak performance of the evaluated test. Indeed, our results match the data in the current literature to a certain extent. A recent meta-analysis suggested that OP swabs should not be recommended for diagnosis of SARS-CoV-2 infection in ambulatory care due to low sensitivity [68% (95% CI 35–94%)] (15), corroborating our results. However, another recent meta-analysis showed that there was no substantial difference between NP and OP swab detection [overall positive detection 88% (95% CI 73–98%) vs. 84% (95% CI 57–100%)] (16). Direct comparison with our findings could hardly be performed, as the latter meta-analysis included both inpatient and outpatient settings, as opposed to our study population that included outpatients with most of them having acute infections. Interestingly, the meta-analysis showed limited overall agreement between NP and OP swabs (68% dual positivity), while in our cohort, all samples detected positive by OP collection were also positive by NP sampling. The lower performance of OP NG-Test in our study could be attributed either to the fact that RT-PCR was performed in NP swabs or that the patients enrolled presented during acute infection, showing major discomfort (nausea and intense cough) during the OP sampling.

Several studies, involving different study designs, brands of RADTs, patient populations, sample sizes and COVID-19 incidence, have assessed the performance of RADTs in various healthcare settings, such as EDs (17–21) and primary healthcare centers (21, 22), showing overall sensitivities ranging from 70.6 to 93.9%. Among the evaluations in the clinical context of a busy ED, a study assessed the performance of a fluorescence immunochromatographic SARS-CoV-2 antigen test (Bioeasy Biotechnology Co., Shenzhen, China), reporting overall sensitivity 93.9% (95% CI 86.5–97.4%) and specificity 100% (95% CI 92.1–100%) (20). The discordant sensitivity compared to our results is probably due to the rather small sample size (*n* = 127), the fact that the RADT was performed by NP and OP swabs placed together in a universal transport medium, the low (9%) local prevalence during the study period and the detection of fluorescence by an instrument in that assay, compared with visual inspection in the NG-Test. Two studies, performed in both symptomatic and asymptomatic patients, evaluated the performance of the STANDARD Q COVID-19 Ag Test (SD BIOSENSOR, KR) (19, 20). The first study reported overall sensitivity 82.9% (95% CI 81–84.8%) and overall specificity 99.1% (95% CI 98.8–99.3%) (19), but the sample type used for antigen testing is not mentioned. The second study evaluated NP samples and showed overall sensitivity 70.6%, specificity 100%, PPV 100%, and NPV 87.4% (20), which are in agreement with our findings. The performance of RADTs assessed in two more studies in adult EDs, the first by deep oro-nasopharyngeal swabs and the latter by not clear sampling, was similar: sensitivity 75.3 and 77.9%; specificity 100 and 98.1%; PPV 100 and 97.3%; NPV 89.2 and 84%, respectively (17, 21), corroborating our results even though clinical samples of strictly symptomatic patients were tested, as opposed to our cohort (8% asymptomatic).

In accordance with field evaluations (18, 20–22), and similarly to the performance characteristics of other RADTs (9, 25), the sensitivity of both the NP and OP NG-Test was increased for specimens with high viral loads (C_t_ ≤ 25), indicating that in high prevalence settings such as the EDs, the assay may be used to timely isolate positive patients, most likely contagious (24), especially when performed by NP collection (99% sensitivity). Interestingly, three RT-PCR positive asymptomatic patients with C_t_ values <25, all contacts of confirmed cases and one reporting history of fever, headache, dizziness and diarrhea 7 days prior to the ED presentation, were detected by both NP/OP NG-Test (data not shown), suggesting that the assay could be quite beneficial in high prevalence clinical settings, where the identification of asymptomatic infections is of utmost importance. However, the moderate NPVs should also be kept in mind when interpreting the test results. Therefore, the medical history and clinical data should also be taken into account and confirmatory RT-PCR testing should be conducted in patients with high clinical suspicion of COVID-19 in high prevalence settings.

It is noteworthy that our study was carried out while B.1.1.7 was the predominant variant in Greece. As the assay under evaluation detects the nucleocapsid protein of SARS-CoV-2, which is conserved, possible new emerging virus variants with mutations in spike but conserved nucleocapsid protein, may not affect its performance.

The single-center nature of the present study could be considered as a limitation. Nevertheless, “Attikon” hospital serves as the referral COVID-19 diagnostic center for the 2nd Regional Health Authority, which comprises 23 hospitals and 267 primary healthcare structures located in Western and Southern Attica, Piraeus and the Aegean islands. The use of a single large testing center allowed standardization as well as increased quality in sample and data collection. Regarding RT-PCR, the same kits and instruments were used leading to standardized results and C_t_ values. Of note, our study was carried out within a population with high prevalence of COVID-19 since the highest burden of SARS-CoV-2 infections in the Athens metropolitan area occurred in Piraeus and West Attica, as recently described (26) and, indeed, 51% of patients tested were RT-PCR positive. Lastly, our sample included mainly (92%) symptomatic but also asymptomatic (8%) individuals, which were not excluded since the scope of our study was focused on the appropriateness of NG-Test in a real-life setting where symptomatic population represents 75% of COVID-19 cases in Greece (23).

## Conclusion

Taken together, the performance of the NG-Test showed overall sensitivity of 81% for NP and 51% for OP samples, but high specificity (99–100%) and PPV (99–100%) regardless of the clinical specimen. The NP NG-Test could be used as a valuable diagnostic tool for the qualitative detection of SARS-CoV-2 in patients with acute infection given its high sensitivity (99%) in samples with high viral load (C_t_ ≤ 25). False-negative antigen test results have been identified, specifically in samples with lower viral loads. Therefore, the medical history and clinical data should also be considered when interpreting the test results and confirmatory RT-PCR testing may be conducted in selected patients with high clinical suspicion of COVID-19 and having negative NP NG-Test result, while patients with positive NG-Test do not require RT-PCR.

## Data Availability Statement

The raw data supporting the conclusions of this article will be made available by the authors, without undue reservation.

## Author Contributions

All authors listed have made a substantial, direct, and intellectual contribution to the work and approved it for publication.

## Funding

This study received funding from NG Biotech, which was not involved in the study design, collection, interpretation of data, the writing of this article or the decision to submit this manuscript for publication.

## Conflict of Interest

The authors declare that the research was conducted in the absence of any commercial or financial relationships that could be construed as a potential conflict of interest.

## Publisher's Note

All claims expressed in this article are solely those of the authors and do not necessarily represent those of their affiliated organizations, or those of the publisher, the editors and the reviewers. Any product that may be evaluated in this article, or claim that may be made by its manufacturer, is not guaranteed or endorsed by the publisher.

## Abbreviations

ED: Emergency Department
NP: nasopharyngeal
OP: oropharyngeal
NG-Test: NG-Test^®^ SARS-CoV-2 Ag
RADT: rapid antigen detection test.

## References

[1] Phelan AL Katz R Gostin LO. The novel Coronavirus originating in Wuhan, China: challenges for global health governance. JAMA. (2020) 323:709–10. 10.1001/jama.2020.1097 31999307

[2] World Health Organization. Coronavirus Disease (COVID-19). (2021). Available online at: https://www.who.int/emergencies/diseases/novel-coronavirus-2019 (accessed December 8, 2021).

[3] Eurosurveillance Editorial Team. Rapid risk assessment from ECDC: resurgence of reported cases of COVID-19 in the EU/EEA, the UK and EU candidate and potential candidate countries. Euro Surveill. (2020) 25:2007021. 10.2807/1560-7917.ES.2020.25.26.2007021 32643600 PMC7346365

[4] Aiyar Y Chandru V Chatterjee M Desai S Fernandez A Gupta A. India's resurgence of COVID-19: urgent actions needed. Lancet. (2021) 397:2232–4. 10.1016/S0140-6736(21)01202-2 34048696 PMC8148651

[5] CDC. CDC 2019-Novel Coronavirus (2019-nCoV) - Real-Time RT-PCR Diagnostic Panel - Catalog #2019-nCoVEUA-01 1000 Reactions. (2019). Available online at: https://www.fda.gov/media/134922/download (accessed December 8, 2021).

[6] Younes N Al-Sadeq DW AL-Jighefee H Younes S Al-Jamal O Daas HI. Challenges in laboratory diagnosis of the novel Coronavirus SARS-CoV-2. Viruses. (2020) 12:582. 10.3390/v12060582 32466458 PMC7354519

[7] Dinnes J Deeks JJ Berhane S Taylor M Adriano A Davenport C. Rapid, point-of-care antigen and molecular-based tests for diagnosis of SARS-CoV-2 infection. Cochrane Database Syst Rev. (2021) 3:CD013705. 10.1002/14651858.CD013705.pub2 33760236 PMC8078597

[8] World Health Organization. Antigen-Detection in the Diagnosis of SARS-CoV-2 Infection: Interim Guidance. (2021). Available online at: https://apps.who.int/iris/handle/10665/345948 (accessed December 8, 2021).

[9] Krüttgen A Cornelissen CG Dreher M Hornef MW Imöhl M Kleines M. Comparison of the SARS-CoV-2 Rapid antigen test to the real star Sars-CoV-2 RT PCR kit. J Virol Methods. (2021) 288:114024. 10.1016/j.jviromet.2020.114024 33227341 PMC7678421

[10] Aranha C Patel V Bhor V Gogoi D. Cycle threshold values in RT-PCR to determine dynamics of SARS-CoV-2 viral load: An approach to reduce the isolation period for COVID-19 patients. J Med Virol. (2021) 93:6794-7. 10.1002/jmv.27206 34264527 PMC8426941

[11] Callaway E. Heavily mutated Omicron variant puts scientists on alert. Nature. (2021) 600:21. 10.1038/d41586-021-03552-w 34824381

[12] Ranganathan P Aggarwal R. Common pitfalls in statistical analysis: understanding the properties of diagnostic tests - Part 1. Perspect Clin Res. (2018) 9:40–3. 10.4103/picr.PICR_170_17 29430417 PMC5799952

[13] NG Biotech. NG-TEST^®^ COVID-19. (2021). Available online at: https://ngtest-covid-19.com/NG-Test-sars-cov-2-ag-cassette/ (accessed February 7, 2022).

[14] Fourati S Langendorf C Audureau E Challine D Michel J Soulier A. Performance of six rapid diagnostic tests for SARS-CoV-2 antigen detection and implications for practical use. J Clin Virol. (2021) 142:104930. 10.1016/j.jcv.2021.104930 34390929 PMC8310570

[15] Tsang NNY So HC Ng KY Cowling BJ Leung GM Ip DKM. Diagnostic performance of different sampling approaches for SARS-CoV-2 RT-PCR testing: a systematic review and meta-analysis. Lancet Infect Dis. (2021) 21:1233–45. 10.1016/S1473-3099(21)00146-8 33857405 PMC8041361

[16] Lee RA Herigon JC Benedetti A Pollock NR Denkinger CM. Performance of saliva, oropharyngeal swabs, and nasal swabs for SARS-CoV-2 molecular detection: a systematic review and meta-analysis. J Clin Microbiol. (2021) 59:e02881-20. 10.1128/JCM.02881-20 33504593 PMC8091856

[17] Möckel M Corman VM Stegemann MS Hofmann J Stein A Jones TC. SARS-CoV-2 antigen rapid immunoassay for diagnosis of COVID-19 in the emergency department. Biomarkers. (2021) 26:213–20. 10.1080/1354750X.2021.1876769 33455451 PMC7898296

[18] Porte L Legarraga P Vollrath V Aguilera X Munita JM Araos R. Evaluation of a novel antigen-based rapid detection test for the diagnosis of SARS-CoV-2 in respiratory samples. Int J Infect Dis. (2020) 99:328–33. 10.1016/j.ijid.2020.05.098 32497809 PMC7263236

[19] Turcato G Zaboli A Pfeifer N Sibilio S Tezza G Bonora A. Rapid antigen test to identify COVID-19 infected patients with and without symptoms admitted to the Emergency Department. Am J Emerg Med. (2021) 51:92–7. 10.1016/j.ajem.2021.10.022 34717211 PMC8530784

[20] Cerutti F Burdino E Milia MG Allice T Gregori G Bruzzone B. Urgent need of rapid tests for SARS CoV-2 antigen detection: evaluation of the SD-Biosensor antigen test for SARS-CoV-2. J Clin Virol. (2020) 132:104654. 10.1016/j.jcv.2020.104654 33053494 PMC7522649

[21] Thell R Kallab V Weinhappel W Mueckstein W Heschl L Heschl M. Evaluation of a novel, rapid antigen detection test for the diagnosis of SARS-CoV-2. PLoS One. (2021) 16:e0259527. 10.1371/journal.pone.0259527 34843505 PMC8629250

[22] Albert E Torres I Bueno F Huntley D Molla E Fernández-Fuentes MÁ. Field evaluation of a rapid antigen test (Panbio™ COVID-19 Ag Rapid Test Device) for COVID-19 diagnosis in primary healthcare centres. Clin Microbiol Infect. (2021) 27:472.e7–10. 10.1016/j.cmi.2020.11.004 33189872 PMC7662075

[23] Brümmer LE Katzenschlager S. Gaeddert M Erdmann C Schmitz S Bota M. Accuracy of novel antigen rapid diagnostics for SARS-CoV-2: a living systematic review and meta-analysis. PLoS Med. (2021) 18:e1003735. 10.1371/journal.pmed.1003735 34383750 PMC8389849

[24] Routsias JG Mavrouli M Tsoplou P Dioikitopoulou K Tsakris A. Diagnostic performance of rapid antigen tests (RATs) for SARS-CoV-2 and their efficacy in monitoring the infectiousness of COVID-19 patients. Sci Rep. (2021) 11:22863. 10.1038/s41598-021-02197-z 34819567 PMC8613285

[25] Maltezou HC Krumbholz B Mavrouli M Tseroni M Gamaletsou MN Botsa E. A study of the evolution of the third COVID-19 pandemic wave in the Athens metropolitan area, Greece, through two cross-sectional seroepidemiological surveys: March, June 2021. J Med Virol. 94:1465–72. (2021). 10.1002/jmv.27465 34812522 PMC9011894

[26] Maltezou HC Papadima K Gkolfinopoulou K Ferentinos G Mouratidou E Andreopoulou A. Coronavirus disease 2019 pandemic in Greece, February 26 - May 3, 2020: the first wave. Travel Med Infect Dis. (2021) 41:102051. 10.1016/j.tmaid.2021.102051 33819570 PMC8016712

